# Classification of Aflatoxin B1 Concentration of Single Maize Kernel Based on Near-Infrared Hyperspectral Imaging and Feature Selection

**DOI:** 10.3390/s21134257

**Published:** 2021-06-22

**Authors:** Quan Zhou, Wenqian Huang, Dong Liang, Xi Tian

**Affiliations:** 1 National Engineering Research Center for Agro-Ecological Big Data Analysis & Application, Anhui University, Hefei 230601, China; p18101007@stu.ahu.edu.cn; 2 Beijing Research Center of Intelligent Equipment for Agriculture, Beijing 100097, China; huangwq@nercita.org.cn (W.H.); tianx@nercita.org.cn (X.T.)

**Keywords:** hyperspectral image, maize kernel, aflatoxin B1, key wavelength selection, dimensionality reduction

## Abstract

A rapid and nondestructive method is greatly important for the classification of aflatoxin B1 (AFB1) concentration of single maize kernel to satisfy the ever-growing needs of consumers for food safety. A novel method for classification of AFB1 concentration of single maize kernel was developed on the basis of the near-infrared (NIR) hyperspectral imaging (1100–2000 nm). Four groups of AFB1 samples with different concentrations (10, 20, 50, and 100 ppb) and one group of control samples were prepared, which were preprocessed with Savitzky–Golay (SG) smoothing and first derivative (FD) algorithms for their raw NIR spectra. A key wavelength selection method, combining the variance and order of average spectral intensity, was proposed on the basis of pretreated spectra. Moreover, principal component analysis (PCA) was conducted to reduce the dimensionality of hyperspectral data. Finally, a classification model for AFB1 concentrations was developed through linear discriminant analysis (LDA), combined with five key wavelengths and the first three PCs. The results show that the proposed method achieved an ideal performance for classifying AFB1 concentrations in a single maize kernel with overall accuracy, with an F1-score and Kappa values of 95.56%, 0.9554, and 0.9444, respectively, as well as the test accuracy yield of 88.67% for independent validation samples. The combinations of variance and order of average spectral intensity can be used for key wavelength selection which, combined with PCA, can achieve an ideal dimensionality reduction effect for model development. The findings of this study have positive significance for the classification of AFB1 concentration of maize kernels.

## 1. Introduction

Maize is one of the most important food crops in the world, but it is vulnerable to fungus infection (e.g., flavus infection) in unsuitable storage conditions. Aflatoxin is a secondary metabolite of some species of Aspergillus fungi (e.g., A. flavus and A. parasiticus), which has been listed as ‘I’ carcinogen by the International Agency for Research on Cancer (IARC) due to its carcinogenicity and mutagenicity [1]. Aflatoxin can be divided into AFB1 (C17H12O6), AFB2 (C17H14O6), AFG1 (C17H12O7), AFG2 (C17H14O7), and approximately 20 other subtypes, among which AFB1 is the most common and toxic, therefore its contents in food are strictly limited in many countries [2]. The limited standards of China are 20 μg/kg (ppb) and 100 ppb in food and feed grades, respectively. The Food and Drug Administration of the United States limits the highest level of 20 ppb AFB1 for human food and products [3].

In the previous decades, AFB1 was detected mainly by thin layer chromatography (TLC), high-performance liquid chromatography (HPLC), and enzyme-linked immunosorbent assay; although these methods are accurate, their instruments are complex and detection speed is slow. In other words, these methods mentioned above could not meet the needs for a rapid nondestructive method in modern industry, as they are inefficient for a large of samples.

As a rapid and nondestructive technology, hyperspectral imaging technology integrates image information and spectral information into one system, which could record spatial and spectral information of given samples simultaneously [4,5]. At present, hyperspectral imaging technology has been used to detect the aflatoxin concentration of crops. On the basis of hyperspectral imaging, Han et al. [6] proposed a convolutional neural network (CNN) method to detect aflatoxin in peanuts, and obtained an overall recognition rate above 95% on a pixel-level and above 90% on a kernel level. Jiang et al. [7] collected the near-infrared (NIR) hyperspectral images between 970 and 2570 nm of peanut kernels and judged whether peanut kernels were moldy by combining PCA and threshold segmentation method, achieving a test accuracy of 87.14%. Bertani et al. [8] developed a classification model of slurried almonds with support vector machine (SVM) algorithm based on the fluorescence spectra induced by specific wavelength; the classification accuracy exceeded 94% for low and high concentrations of AFB (AFB1 + AFB2). These results illustrated the feasibility of using hyperspectral imaging technology to detect aflatoxin in food. In terms of the classification of AFB1 concentration of maize kernels, Chu et al. [9] collected the hyperspectral images of maize kernels contaminated by AFB1 using the short-wave infrared hyperspectral imaging system, a qualitative classification model of AFB1 concentration was developed by combining the first five principal components (PCs) with SVM algorithm. Zhu et al. [10] proposed the integration of fluorescence and reflectance visible/near infrared (VNIR) hyperspectral images to detect aflatoxins in maize kernels. PCA and a least squares support vector machine (LS-SVM) were used to classify contaminated and healthy kernels, with the overall prediction accuracy of 95.33%. Tao et al. [11] established the classification models of maize kernels with different AFB1 concentrations with 410–1070 nm and 1120–2470 nm spectra, respectively, and the results showed that the effect of using NIR spectra was better than that of VNIR bands. Chakraborty et al. [12] classified maize kernels with six different concentrations of AFB1, using hyperspectral images at 400–1000 nm, and the study showed that partial least squares discriminant analysis (PLS-DA) could achieve good results under 12 Latent Variables (LVs). Kimuli et al. [13] investigated the feasibility of detecting AFB1 on the surface of maize kernels with a VNIR hyperspectral imaging system. A combination of PCA and factor discriminant analysis (FDA) was used and achieved a good discriminant result. These studies promoted the development nondestructive detection technology in AFB1 concentration detection of maize kernels. However, the recognition accuracies in the test of Chu et al. [9] were low. Zhu et al. [10] achieved high classification accuracy, but only two concentrations classification were studied; the concentration classes were too few to carry out a more detailed classification. Tao et al. [11] and Chakraborty et al. [12] studied the differentiation of multiple concentration levels; Kimuli et al. [13] discussed the sample differences of four different regions of origin, but their research samples specific to a certain concentration are too few to be widely used.

Linear discriminant analysis (LDA) is a classic classification algorithm, which is widely used in the field of pattern recognition and image processing. Nowadays, LDA has been successfully used in the classification of hyperspectral data. Li et al. [14] proposed a new method to analyze the age of blood stains using hyperspectral image. LDA was applied, and achieved an ideal effect. Garcia-Allende et al. [15] detected spurious elements in raw material processing chains on the basis of the hyperspectral image of materials, and identified the components in tobacco with LDA, achieving a good recognition effect. On the basis of hyperspectral images of 17 varieties of maize kernels, Xia et al. [16] classified the variety of maize kernel with successive projection algorithm (SPA) coupled with LDA, achieving an accuracy of 90.31%. These studies show that LDA can be applied to classify hyperspectral data with good results in some application fields. This study explored the application effect of LDA, coupled with NIR hyperspectral imaging, in AFB1 concentration detection of single maize kernels.

The hyperspectral image information is huge and redundant within adjoining wavelengths; it is needed to compress data from high dimensionality to low dimensionality for improving detection efficiency using variable selection algorithms. Previous studies on this aspect often reduced the dimensionality of hyperspectral image with a dimensionality reduction algorithm, and then achieved the classification of different concentrations of AFB1 samples combined with the classification model. In this study, the dimensionality reduction in hyperspectral data was discussed, and a new key wavelength selection method was proposed by considering the between-class variance and order of average spectral intensity. A new attempt was carried out on combining this method with dimensionality reduction algorithm for compressing hyperspectral data; the result was better than that of using dimensionality reduction or characteristic wavelength alone, and the efficient compression of hyperspectral data is realized.

The present study aims to investigate the feasibility of using the combination of dimensionality reduction and key wavelengths to obtain the best modeling data in the NIR region of 1100–2000 nm, combined with classification model to detect AFB1 concentration in maize kernels. The specific objectives of this study were to: (1) compare the differences between raw and pretreated spectra of different AFB1 concentrations of maize kernels and analyze the effects of pretreatment; (2) select the optimal dimensionality reduction algorithm for this study through analysis and comparison of different dimensionality reduction methods; (3) select key wavelengths according to spectral features, such as the variance and order of average spectral intensity, and identify the optimal data for modeling through the combination of specific wavelength and dimensionality reduction algorithm; and (4) obtain an optimal calibration model by comparing different classification models based on low-dimensional data.

## 2. Materials and Methods

### 2.1. Sample Preparation

The variety of maize kernel in this study is Zhengdan 958, which is widely planted in China. All samples were provided by Beijing Academy of agriculture and Forestry Sciences, and were planted in Xiaotangshan, Beijing, North China, one of the main maize producing areas in China. Therefore, the research object of this study is representative. A total of 450 maize kernels without any defect were selected to make the maize kernel samples, with five different concentrations of AFB1. In order to ensure that there were no pre-existing natural toxins, 20 representative kernels were randomly selected from each group and the samples were tested as toxin-free by Beijing Putian Tongchuang Biotechnology Co., Ltd. In order to prepare the maize samples with five different concentrations of AFB1 artificially (0, 10, 20, 50, and 100 ppb), five different concentrations of AFB1 diluent were prepared according to the average kernel weight (about 0.5 g).

The diluent and samples were prepared as follows: initially, 5 mL of AFB1 toxin (2 μg/mL), 5 mL of 100% methanol, and other necessary equipment were prepared. Then, 0.2 μg ml^−1^ diluent was created by mixing 0.25 mL of solution of toxin with 2.25 mL of methanol, and 90 samples with 10 ppb AFB1 contents were prepared by depositing 25 μL diluent on each embryoid side of each kernel (25 μL × 0.2 μg/mL = 0.005 μg of toxin per 0.5 g kernel). To achieve 20 ppb kernel samples, 0.4 μg/mL diluent was created by mixing 0.5 mL of solution of toxin with 2 mL of methanol, and 25 μL of diluent was deposited on each kernel to achieve 20 ppb (25 μL × 0.4 μg/mL = 0.01 μg of toxin per 0.5 g kernel). Following a similar procedure, different concentrations of diluents were prepared by changing the mixture ratio of toxin and methanol. Then, the diluents of different concentrations were dropped on the kernels to achieve 50 and 100 ppb. Each sample in the control group was treated with a 25 μL methanol-alone drop to eliminate the effect of diluent treatment as much as possible. After these kernels were inoculated, they were placed in a chemical hood to allow the diluent to dry. Then, after 120 min, the methanol in these samples volatilized, and AFB1 was absorbed completely by the samples [17,18,19]. All samples were prepared and could be used for spectral collection.

### 2.2. Hyperspectral Imaging System

**Figure 1:**
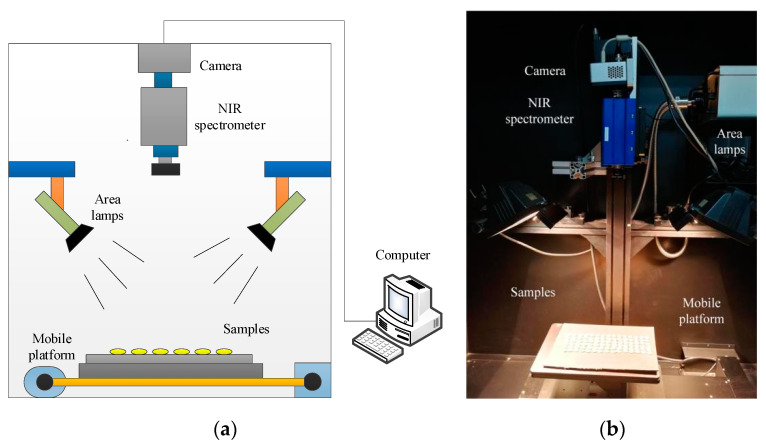
Hyperspectral imaging system. (**a**) Block diagram for HIS; (**b**) Real laboratory system.

A line-scanning NIR hyperspectral imaging system (Figure 1) was used to acquire the hyperspectral images of the samples. The system consists of a 14-bit NIR charge-coupled device camera (Xeva−2.5−320, Xenics Ltd., Leuven, Belgium), a high-resolution spectrometer covering 930–2548 nm with an interval of 6.32 nm (ImSpector N25E, Spectral Imaging Ltd., Oulu, Finland), two 300 W halogen light area sources (Antefore International Co., Ltd., Taiwan, China), a precision mobile platform (EZHR17EN, AllMotion, Inc., Union City, CA, USA), and a computer (Dell, Intel (R) Core (TM) i5-2400 CPU @ 3.10 GHZ).

In order to acquire the high-quality hyperspectral images, the distance between the lens and the sample stage was set at 380 mm, and two halogen light area sources were mounted at 45° angles from horizontal to avoid oversaturation. The movement speed of the sample and the exposure time of camera were set at 42 mm/s and 2 ms, respectively, to avoid image spatial deformation. In order to improve the image collection efficiency, 90 samples with the same AFB1 concentrations (15 rows and 6 columns) were placed on the transport platform and scanned line by line each time. Each NIR hyperspectral image consists of 256 congruent subimages, with wavelengths ranging from 930 nm to 2548 nm. Due to the severe noise in the spectrum before 1100 nm and after 2000 nm, only the spectral range covering 1100–2000 nm (145 wavelengths) was used for the NIR imaging modes to minimize the effect of noise [20].

### 2.3. Hyperspectral Image Correction

The spatial intensity variation in the plane of the scene produced by halogen lamps and the dark current in the CCD camera generally led to the signal of the camera chip to be nonzero when no light hit the detectors and resulted in a large amount of noise in some bands [20]. Therefore, white and dark references were used to correct the raw hyperspectral images to eliminate these adverse interruptions for further analysis. The white reference image was obtained using a white diffuse reflectance board under the same system parameters. The dark reference image was obtained by covering the lens and turning off the lamps [21]. The raw image data were corrected as follows:

(1) $$D=\frac{D_{\mathrm{raw}}-D_{\mathrm{dark}}}{D_{\mathrm{white}}-D_{\mathrm{dark}}}$$

In Equation (1), $D_{\mathrm{raw}}$ represents the original uncorrected hyperspectral image, $D_{\mathrm{dark}}$ represents the dark reference, $D_{\mathrm{white}}$ represents the white reference, and D represents the calibrated hyperspectral image.

### 2.4. Spectral Data Extraction

A hyperspectral image consists of image information and spectra information. In order to develop a maize kernel classification model with different concentrations of AFB1, the image and spectra information of maize kernel were extracted as follows:

Step 1. The grayscale image at 1210.3 nm was selected to segment the maize kernel from the background due to their obvious intensity difference;

Step 2. Threshold segmentation and morphological filtering were employed to remove the background and edge region of the maize samples while remaining in the effective region at the grayscale image at 1210.3 nm;

Step 3. The filtered image was used as the region of interest (ROI) of the sample to mask the original hyperspectral image and extracted the region of each kernel;

Step 4. The spectra data of each kernel within the ROI were averaged at each wavelength, and the average spectra were used for further analysis.

### 2.5. Spectral Pretreatment

The hyperspectral data also contained some disturbances (e.g., external stray light or scattering noise generated by the camera) during the NIR spectra acquisition. In order to reduce the influence of nontarget factors and to establish a stable model, the raw spectra must be pretreated before further analysis [22]. Savitzky–Golay (SG) smoothing was used to reduce the influence of noise from environment and instrumental fluctuations [23]. After SG smoothing, two spectral pretreatment algorithms, including first derivative (FD) and second derivative (SD), were used to preprocess the spectra to highlight the differences between different AFB1 [24].

### 2.6. Key Wavelength Selection

Variable selection methods are usually used to select the key wavelength. Variance could reflect the differences between groups; in this study the key wavelengths used for the classification of AFB1 content in maize kernels were determined by combining the variance of spectral intensity with their order. The specific method is as follows:

Step 1. Calculated the average spectrum of each group;

Step 2. Ordered the average spectral intensity of five groups at each band, and divided the bands with the same order into the same subset. In order to eliminate the effect of tiny intensity difference on ordering, the spectral intensity of adjacent groups would be regarded as equal if their difference was less than 100 times of variance;

Step 3. Calculated the variance of spectral intensity for each band, and only the wavelength with the largest variance was retained in the same subset; the remaining wavelengths were removed;

Step 4. All the remaining wavelengths were sorted according to the variance from large to small, and the first few retained wavelengths were the final selected characteristic wavelengths.

**Figure 2:**
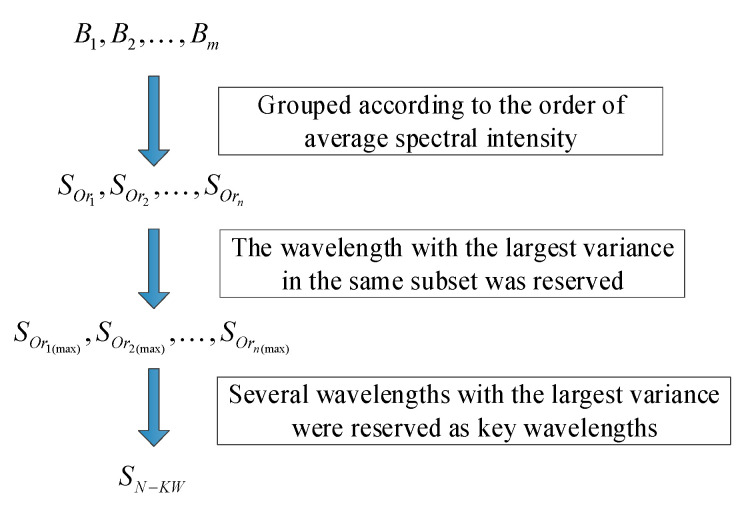
Flowchart of key wavelengths selection in this study.

The flowchart of key wavelength selection in this study is shown in Figure 2. $B_{i}$ represents the i-th wavelengths, and m is the total number of all wavelengths. $S_{\mathrm{or}}$ is the wavelength subset sorted according to the second step, and n is the number of subsets. $S_{\mathrm{or}\left(\max\right)}$ is the remaining wavelength after the third step. $S_{N-\mathrm{KW}}$ is the set of key wavelengths selected after the fourth step, and N is the number of selected key wavelengths.

### 2.7. PCA for Dimensionality Reduction

PCA is a well-known algorithm of multivariate analysis [25]. The main goal of PCA is to reduce the dimensionality of a data set with a large number of inter-correlated variables, while retaining as much of the information that is present in the data as possible. It is commonly used for data dimensionality reduction, elimination of multicollinearity, and enhancement of the hyperspectral dataset to simplify feature selection [26,27]. The hyperspectral image is a massive data cube, therefore PCA is promising to reduce the dimensionality of hyperspectral data and improving the detection efficiency of the prediction model.

### 2.8. Model Development

The basic idea of LDA is to compress the dimensionality of feature space and extract the classification information through projecting the high-dimensional data into the optimal discriminant vector space. The projected data have the minimum within-class distance and the maximum between-class distance in the new subspace, that is, the pattern has the best separability in the new vector space [28].

For a given data set $D={\left\{\left(x_{i},\;y_{i}\right)\right\}}_{i=1}^{m}$, where sample $x_{i}$ is an N-dimensional vector, $y_{i}$ is the label of the i-th sample, and m is the number of samples. ω is the optimal classification vector space, the projection of the centre of the samples on the optimal hyperplane is $\omega^{T}\mu$, where $\mu_{i}$ is the mean vector of class i in the original space vector. ‘Within-class scatter matrix’ and ‘between-class scatter matrix’ are represented as $S_{w}$ and $S_{b}$, respectively. $S_{w}$ and $S_{b}$ are defined in Equations (2) and (3), respectively.

(2) $$S_{w}=\overset{C}{\underset{i=1}{\sum }}\underset{x_{i}^{\left(k\right)}\in X_{i}}{\sum }\left(x_{i}^{\left(k\right)}-\mu_{i}\right){\left(x_{i}^{\left(k\right)}-\mu_{i}\right)}^{T}$$

(3) $$S_{b}=\overset{C}{\underset{i=1}{\sum }}N_{i}\left(\mu_{i}-\mu \right){\left(\mu_{i}-\mu \right)}^{T}$$

In Equations (2) and (3), C is the number of classes of the samples, $N_{i}$ is the number of samples of class i, μ is the mean vector of all samples, $x_{i}^{\left(k\right)}$ is the k-th sample of class i, and $X_{i}$ is the sample of class i.

(4) $$J=\mathrm{argmax}\frac{\omega^{T}S_{b}\omega }{\omega^{T}S_{w}\omega }$$

According to the nature of the ‘Generalised Rayleigh Quotient’, the optimal classification vector space ω can be obtained [29,30]. Then, the class of unknown samples can be predicted according to the optimal vector space.

**Figure 3:**
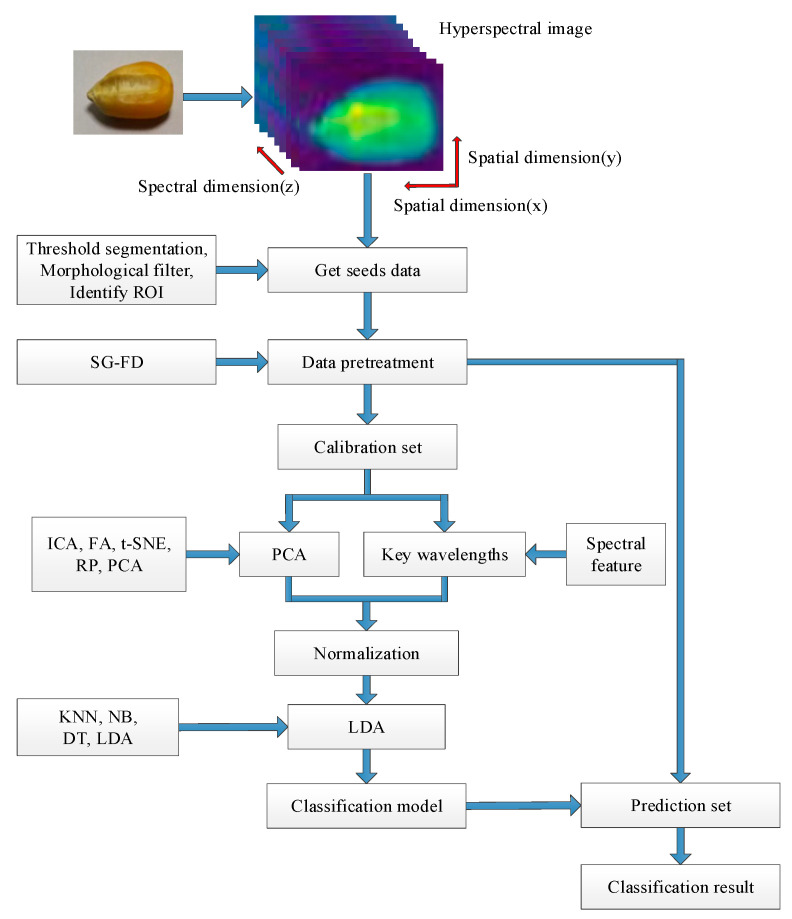
Flowchart of this study.

In this study, there were four groups of samples with different AFB1 contents and one control group of samples without AFB1. All 450 samples (five groups with 90 samples in each group) were randomly divided into the calibration set or prediction set, according to the ratio of 1:1. Thus, 225 samples (45 of each group) were selected as the calibration set to develop the calibration model, and the remaining 225 samples (45 of each group) were used as the prediction set to verify the performance of the developed model. To eliminate the influence of random sample division on the prediction ability of the developed model, the prediction model was developed 10 times by random sample division; the model which came closest to the average performance of the 10 developed models was taken as the final model. To evaluate the classification performance of the proposed method, three parameters, namely accuracy, F1-score, and Kappa, were considered [31,32]. The model development treatments were conducted in the Python 3.7.4 (Guido van Rossum, Netherlands). The flowchart is shown in Figure 3.

## 3. Results

### 3.1. Comparison of Spectral Pretreatment Method

**Figure 4:**
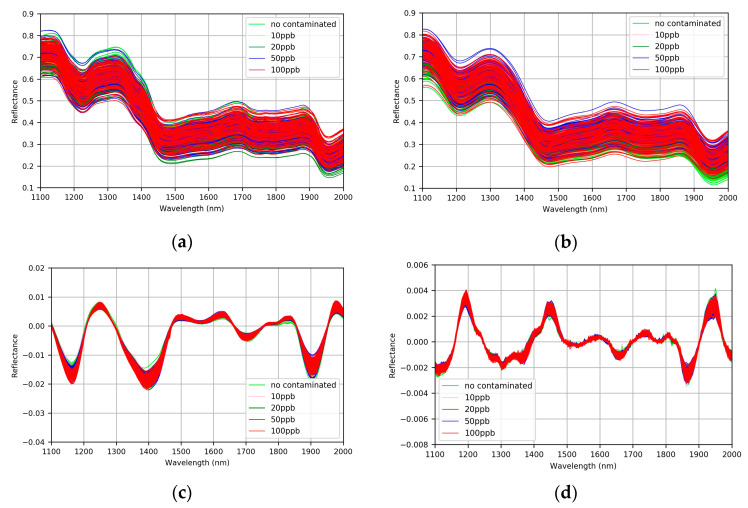
Raw and pretreated spectra of samples in the calibration set (**a**) Raw spectra, (**b**) spectra after SG, (**c**) spectra after SG–FD, and (**d**) spectra after SG–SD.

**Figure 5:**
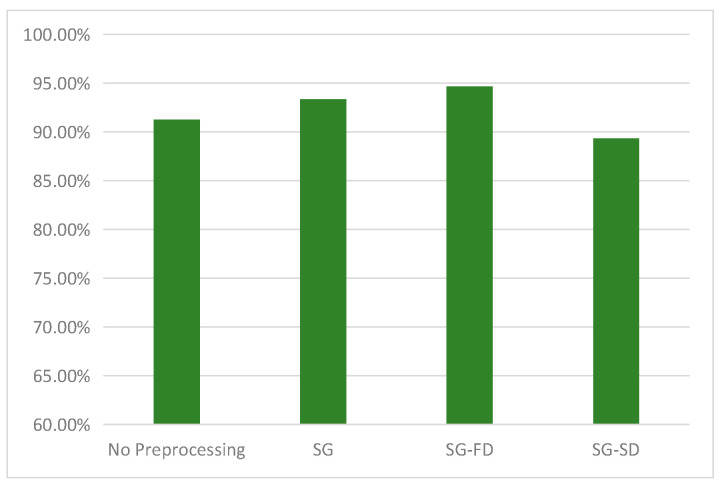
Comparison of the effects of different pretreatment methods on LDA model at full wavelength.

The raw and pretreated spectra of all samples in the calibration set are presented in Figure 4; it could be seen easily that the differences of raw spectra are very small in the between-class, and relatively large in the within-class (Figure 4a). In order to eliminate the noise existing in the raw spectra, firstly SG smoothing of 15 points was employed (Figure 4b). Then, FD and SD were used to further highlight the spectral difference of different AFB1 concentrations [33]. The results show that the spectra pretreated by SD contains a lot of burrs (Figure 4d), while the spectra pretreated by FD shows subtle differences in different AFB1 concentrations (Figure 4c). In order to evaluate the effect of spectral pretreatment methods on the classification for different AFB1 concentrations, the full wavelength spectra pretreated by SD and FD were used to develop the classification models using LDA classifier. The classification results (Figure 5) demonstrate that SG–FD pretreatment is better than that of the others, therefore the pretreated spectra by SG–FD method was used for subsequent analysis.

### 3.2. Results of Key Wavelength Selection

**Figure 6:**
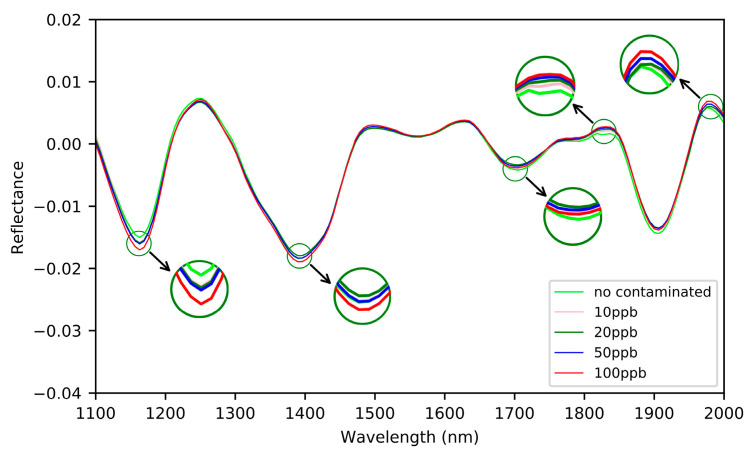
Five key wavelengths selected according to spectral feature.

The key wavelengths were selected according to the variance of average spectral intensity, and their orders are shown in Table 1 (only the first six key wavelengths are shown). V_0_, V_10_, V_20_, V_50_, and V_100_ represent the average spectral values when AFB1 concentration is 0, 10, 20, 50, and 100 ppb at the selected wavelength, respectively. The variance at the first five bands were 4.37, 1.89, 1.83, 1.12, and 1.06, respectively, but the variance decreases rapidly from the sixth wavelength, therefore only the first five wavelengths (1160.5, 1391.8, 1700.9, 1827.9, and 1981.0 nm) were selected as key wavelengths according to our proposed method (Figure 6).

The reflected spectra are related to some chemical bonds, associated mainly with C–H, O–H, and other functional groups due to hydrogenic stretching, bending, or deformation vibration [34]. According to the analysis of chemical bonds, 1160.5 nm corresponds to the C–H regions, 1391.8 nm contributes to the O–H regions stretching the first overtone, 1700.9 nm corresponds to the C–O regions, 1827.9 nm is ascribed to the C–C1 stretch, and 1981.0 nm is associated with the O–H stretch functional group of water and starch [26,35]. In addition, the selected key wavelengths can also reflect the spectral intensity difference of different AFB1 concentrations, as the variable selection method proposed by this study mainly considered the variance of average spectral intensity. Hence, the selected key wavelengths were mostly located at the peaks or valleys of pretreated spectra (Figure 6).

Kandpal et al. [36] analyzed the spectra of samples with different AFB1 concentrations in the range of 1100–1700 nm; six key wavelengths were found using the beta coefficients of the PLS-DA model, of which 1150 nm is close to one of the key wavelengths in this study (1160.5 nm). Wang et al. [26] analyzed the key wavelengths associated with AFB1 concentration in maize seeds in the range of 1000–2500 nm, of which 1146, 1406, and 1704 nm were close to the 1160.5, 1391.8, and 1700.9 wavelengths selected in this study, respectively. Some key wavelengths selected in this study are basically the same as Wang et al. and Kandpal et al. However, there are also some wavelength inconsistencies. To analyze the reasons, firstly, different pretreatment methods and key wavelength selection methods have a great influence on the selection of key wavelengths. Secondly, different AFB1 concentration levels of the study object also affect the selection of the key wavelengths.

### 3.3. PCA in the NIR Wavelength Ranges

**Figure 7:**
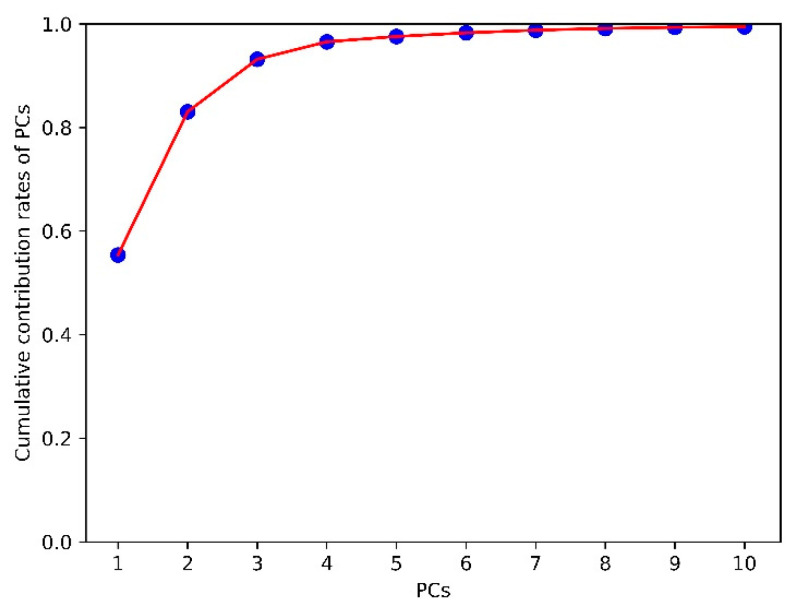
Cumulative contribution rate of the first 10 PCs.

**Figure 8:**
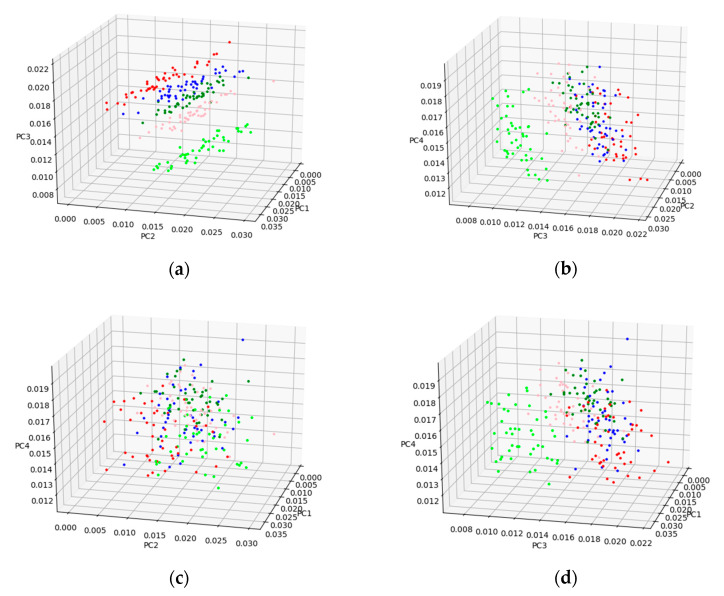
Clustering effect with three PCs (lime green, light pink, green, blue, and red represent 0, 10, 20, 50, and 100 ppb, respectively). (**a**) PC1, PC2, and PC3; (**b**) PC2, PC3, and PC4; (**c**) PC1, PC2, and PC4; and (**d**) PC1, PC3, and PC4.

The cumulative contribution rates can reflect the ability of corresponding PCs to explain the original variables. Figure 7 shows the cumulative contribution rate of the first 10 PCs obtained by PCA, based on the pretreated method of SG-FD. The scores of the first four PC (denoted by PC1, PC2, PC3, and PC4) were 55.37%, 27.67%, 10.13%, and 3.38%, respectively. The contribution rates of the first four PC exceeded 96.5%. The cumulative contribution rate of the fifth and subsequent PCs less than 3.5%. To select the best PCs from the first four PCs for developing a classification model, the clustering effect was used for analysis. For the calibration set samples, the clustering effect of any three combinations of the first four PCs is shown in Figure 8.

As can be seen from Figure 8, the effects of Figure 8a,b were evidently better than that of Figure 8c,d. Thus, PC2 and PC3 contain important information about classification. Moreover, the effect in Figure 8a was better than that in Figure 8b, indicating that PC1 also plays a role in classification. Therefore, the combination of the first three PCs was best for classification of AFB1 concentrations. Figure 8a shows in the first three PC spaces, with a certain degree of discrimination between samples without pollution and samples with contaminated groups, so the samples without contamination can be easily separated from the contaminated groups; the remaining four contaminated groups have some clustering effect, with some overlapping areas that are not fully separated. Therefore, if only the first three PCs are used to establish a classification model, the effect will not be optimal.

### 3.4. Classification Results of AFB1 Level

On the basis of SG–FD pretreated spectra, the first three PCs containing the main classification information were retained. In addition, five key wavelengths were selected according to the combination of variance and size order of average spectra. The normalized first three PCs, five key wavelengths, and the combination of these two datasets were used to replace the full spectra data, respectively. All samples in the prediction set were classified with an LDA classification algorithm; the test accuracies were 91.11%, 79.56%, and 95.56%, respectively. The results show that the combination of these two datasets can improve the classification accuracy compared with the first three PCs or five key wavelengths. Table 2 shows the specific results of AFB1 concentration classification on the prediction set using the combination of these two datasets.

It can be seen from Table 2 that the proposed method obtained an ideal performance, with overall accuracy of 98.22% and 95.56% for the calibration set and prediction set, respectively. For prediction sets, it is worth mentioning that all uncontaminated samples were accurately recognized. However, for the 10 ppb group, one sample was mistaken as uncontaminated (0 ppb) and one was mistaken as 20 ppb. For the 20 ppb group, three samples were misidentified, which were mistaken as 10, 50, and 100 ppb. For the 50 ppb group, one sample was mistaken as 20 ppb, one was mistaken as 100 ppb, and two samples were attributed to 10 ppb. For the 100 ppb group, one sample was mistaken as 20 ppb. It can be seen that most of the misclassified samples were divided into adjacent groups, and the difference of AFB1 concentration between the samples of adjacent groups was not large, which made their spectra very similar, leading to misclassification. In summary, the recognition accuracy of each group was above 90%, with an average accuracy of 95.56%, achieving a relatively ideal result.

### 3.5. Influence of Different Dimensionality Reduction Methods

**Figure 9:**
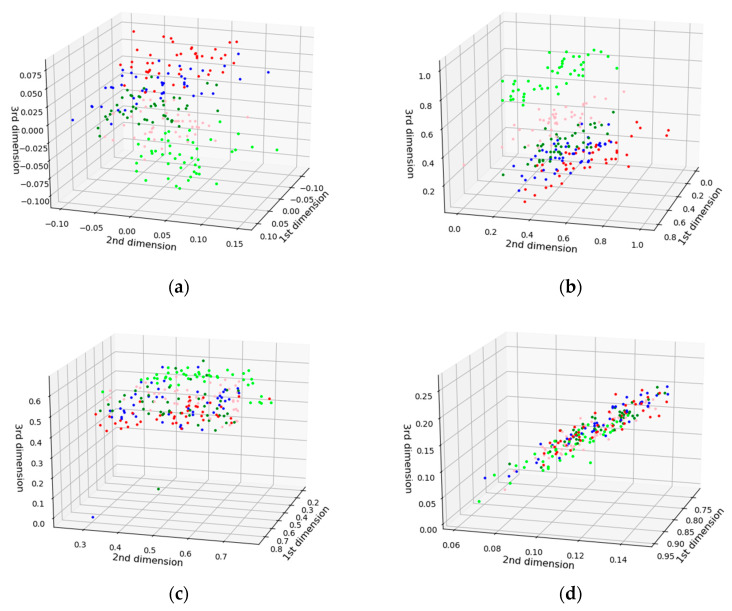
Clustering effect of the first three dimensions of four comparison algorithms (lime green, light pink, green, blue, and red represent 0, 10, 20, 50, and 100 ppb, respectively). (**a**) ICA, (**b**) FA, (**c**) t-SNE, (**d**) RP.

To compare the performance of PCA, four other dimensionality reduction algorithms, including independent component analysis (ICA), factor analysis (FA), t-distributed stochastic neighbor embedding (t-SNE), and random projection (RP), were selected. Figure 8a shows the clustering scatter of the first three PCs of PCA, which proves that the PCA algorithm has a certain effect. The clustering scatter of the four other, different classifiers, based on the first three dimensions, are shown in Figure 9.

It can be seen from Figure 9 that the uncontaminated samples could be well discriminated from contaminated samples by ICA and FA algorithms, however the samples with different AFB1 concentrations could not be distinguished. The t-SNE and RP algorithms achieved poor discrimination performance as all samples were distributed disorderly. Compared with the clustering performance of PCA, all four dimensionality reduction algorithms were worse than that of PCA in this study, which was also confirmed by the classification results of Table 3.

### 3.6. Comparison of Different Classifiers on Discrimination Results

To highlight the superiority of LDA classifier in this study, three other classifiers, including K-nearest neighbors (KNN), Naive Bayes (NB), and Decision Tree (DT), were used to establish the prediction model based on the same dataset, and then their classifier result was compared with LDA [37]. The discrimination results of four different classification models built by four different datasets are shown in Table 4.

Table 4 proves that the classification effect of LDA was better than DT, NB, and KNN in this study. The combinations of the first three PCs and five key wavelengths for classification of AFB1 contents of single maize kernel achieved accuracy, F1-score, and Kappa values of 95.56%, 0.9554, and 0.9444, respectively. The three evaluation parameters of the proposed method were better than full wavelengths, achieving a good classification effect of AFB1 contamination of single maize kernel.

### 3.7. Independent Validation of New Samples

To further verify the effect of this model, 30 new samples in each group were re-prepared with the same method. The hyperspectral images of samples were preprocessed in the same way. Under the LDA model, the spectral data of the five key wavelengths and the first three PCs combinations were used for testing, and the results are shown in Table 5.

It can be seen from Table 5 that, based on the proposed method, AFB1 concentration of new validation samples was classified with the desired results, and the test accuracy of 88.67%, which is slightly lower than that of the prediction set samples due to certain time intervals between the spectra acquisition time of the new samples and the original samples, as well as the variability between days and the readjustment of the hyperspectral acquisition equipment, which may also impact the spectra of samples. This aspect will be considered in future studies.

## 4. Discussion

Table 6 shows the results of the comparison between the proposed method and other researchers’ methods. As shown in Table 6, Tao et al. [11] and Chakraborty et al. [12] used PLS-DA to select 18 and 12 LVs, respectively, and established a classification model, which achieved fair classification results. Kimuli et al. [13] selected 12 PCs of PCA to build their classification model, which achieved an ideal result. Although the above research results were ideal, they were all obtained under relatively high data dimension. Chu et al. [9] retained only 5 PCs, but did not achieve a desired classification effect. Comparatively, this study achieved desirable results on low-dimensional data (8 dimensional data).

Using NIR spectroscopy technology to discriminate the AFB1 concentration of single maize is still in the exploratory stage. It is difficult to control the content of AFB1 produced by natural mold, therefore the artificial inoculation of toxin becomes a simple and effective sample preparation method due to the surface distribution characteristics of AFB1 [38]. Tao et al. [11], Chakraborty et al. [12], and Kimuli et al. [13] also prepared the contaminated maize kernel samples with the same or similar method as this study.

An ideal result for classification of different AFB1 concentration was obtained based on the proposed method of this study. Although the established model has achieved satisfactory results, the success rate for classification of 20 and 50 ppb groups still needs to be improved. Figure 8a shows that there is an overlapping area between 20 ppb and 50 ppb groups and their adjacent groups. In addition, Table 1 shows that the average spectral intensities of 20 ppb and 50 ppb samples were almost the same at three of the five selected key wavelengths. Therefore, it is not difficult to understand why the classification effects of these two groups were slightly poor. It can also be analyzed that the overlapping areas of the other three groups (0, 10, and 100 ppb) in Figure 8a were not as much as those of 20 ppb and 50 ppb, and the spectral intensity difference from the other three groups in the five key wavelengths was also higher than that of those two. Therefore, the classification effects of the three groups were better than that 20 ppb and 50 ppb.

PCA has good dimensionality reduction effect. It can be seen from Figure 8 that the first three PCs contained the main classification information about AFB1 concentrate, and the classification model was fairly good when only the first three PCs were used for modeling. However, it does not mean that increasing the number of PCs could improve the accuracy of classification. In this study, when the number of PCs used for modeling exceeded three, the classification accuracy almost ceased to improve with the increase of PCs. Therefore, the first three PCs were selected in this study, which not only retained the most important classification information, but also made the data dimension as small as possible. The key wavelength selection method proposed in this study takes into account both the variance and order of spectral intensity of different groups simultaneously. The characteristic wavelengths with certain classification ability were combined by limiting a large number of key wavelengths with repeated features, and reserving a few characteristic wavelengths with spectral differences of different groups. However, the result yield by five key wavelengths was not ideal, as the key wavelength was selected according to the variance of average spectral intensity of between-class, and ignored the difference of various spectral intensity of within-class. In this study, the key wavelength selection method we proposed was combined with PCA to establish an effective classification model. The test results on the prediction set and independent verification set show that the scheme in this study is feasible.

## 5. Conclusions

On the basis of the NIR hyperspectral image in the 1100–2000 nm, with pretreatment by SG and FD, five key wavelengths were selected by combining variance and order of spectral intensity, and the data of the first three dimensions were obtained by PCA dimensionality reduction based on full wavelength. Then, LDA was used to establish the classification model based on the data of combinations of five key wavelengths and the first three PCs. The proposed algorithm achieved an ideal result to classify the AFB1 concentrations of maize kernels. Four groups of samples with different AFB1 contents and a group of control samples were selected for this study; 90 samples of each group were divided into the calibration set and the prediction set, according to the ratio of 1:1. The results show that the average test accuracy of AFB1 concentrations of maize kernels in the prediction set was 95.56%, which is better than the results of full wavelengths and other dimensionality reduction methods. For independent validation samples, the test accuracy reached 88.67%. This study has certain reference value for the classification of AFB1 concentrations of single maize kernels. In the future work, different varieties of maize kernels and more accurate detection of AFB1 concentration levels will be studied.

## Figures and Tables

**Table 1:** Variance and order of average spectral intensity at different wavelengths.

Variance (×10^−7^)	4.37	1.89	1.83
Wavelength	1160.5 nm	1827.9 nm	1981.0 nm
Order of size	V_0_ > V_10_ = V_20_ = V_50_ > V_100_	V_100_ = V_50_ = V_20_ > V_10_ > V_0_	V_100_ > V_50_ > V_20_ = V_10_ > V_0_
Variance (×10^−7^)	1.12	1.06	0.45
Wavelength	1391.8 nm	1700.9 nm	1898.0 nm
Order of size	V_20_ > V_0_ = V_10_ = V_50_ > V_100_	V_20_ = V_10_ = V_50_ > V_100_ > V_0_	V_50_ = V_100_ = V_20_ = V_10_ > V_0_

**Table 2:** Confusion matrix of the predicted results of AFB1 contents.

Data Set	Real AFB1 Contents	Predicted Results						
		0 ppb	10 ppb	20 ppb	50 ppb	100 ppb	Accuracy	Overall Accuracy
Calibration set	0 ppb	45	0	0	0	0	100%	98.22%
	10 ppb	0	44	1	0	0	97.78%	
	20 ppb	0	1	43	1	0	95.56%	
	50 ppb	0	1	0	44	0	97.78%	
	100 ppb	0	0	0	0	45	100%	
Prediction set	0 ppb	45	0	0	0	0	100%	95.56%
	10 ppb	1	43	1	0	0	95.56%	
	20 ppb	0	1	42	1	1	93.33%	
	50 ppb	0	2	1	41	1	91.11%	
	100 ppb	0	0	1	0	44	97.78%	

**Table 3:** Comparison the effect of different dimensionality reduction algorithms.

Pretretment	Classification Method	Dimensionality Reduction Algorithms	Dimensions	Classification Results (*/**)					Accuracy
				0	10	20	50	100	
SG-FD	LDA	PCA	3	45/45	42/45	38/45	37/45	43/45	91.11%
		ICA	3	43/45	41/45	31/45	24/45	36/45	77.78%
		FA	3	44/45	43/45	35/45	25/45	41/45	83.56%
		t-SNE	3	40/45	10/45	13/45	6/45	29/45	43.56%
		RP	3	37/45	9/45	15/45	13/45	28/45	45.33%

* represents the number of accurately predicted samples in the prediction set. ** represents the total number of samples in the prediction set.

**Table 4:** Discrimination results of different classification models.

Pretretment	Wavelengths or Dimensions	Number of Wavelengths or Dimensions	Accuracy/F1-Score/Kappa			
			LDA	KNN	NB	DT
SG-FD	Full wavelength	145	94.67% 0.9466 0.9333	69.33% 0.6951 0.6166	80.89% 0.8076 0.7611	91.56% 0.9149 0.8944
	PCs	3	91.11% 0.9094 0.8889	66.67% 0.6610 0.5833	72.89% 0.7242 0.6611	74.22% 0.7397 0.6778
	Key wavelengths	5	79.56% 0.7912 0.7444	52.89% 0.5260 0.4111	49.33% 0.4942 0.3667	70.22% 0.7053 0.6278
	PCs + key wavelengths	8	95.56% 0.9554 0.9444	74.67% 0.7432 0.6833	80.44% 0.8028 0.7556	82.67% 0.8265 0.7833

**Table 5:** Test results of independent validation of new samples.

Data Set	Real AFB1 Contents	Predicted Results						
		0 ppb	10 ppb	20 ppb	50 ppb	100 ppb	Accuracy	Overall Accuracy
Independent validation samples	0 ppb	29	1	0	0	0	96.67%	88.67%
	10 ppb	1	27	2	0	0	90.00%	
	20 ppb	0	2	25	2	1	83.33%	
	50 ppb	0	1	3	25	1	83.33%	
	100 ppb	0	0	1	2	27	90.00%	

**Table 6:** Comparison of the results with others.

Methods	Wavelength Range	AFB1 Concentration Level	Principal Method	Dimensions of Modeling Data	Accuracy of Prediction Set
Proposed method	1100–2000 nm	0, 10, 20, 50, and 100 ppb	PCA + key wavelengths LDA	3PCs + 5 key wavelengths	95.56% and 88.67%
Tao et al. [11]	410–1070 nm and 1120–2470 nm	0, 10, 20, 50, 100, 500, and 1000 ppb	PLS-DA	18 LVs	91.4% and 97.1%
Chakraborty et al. [12]	400–1000 nm	25, 40, 70, 200, 300, and 500 ppb	PLS-DA	12 LVs	94.7%
Kimuli et al. [13]	400–1000 nm	0, 10, 20, 100, and 500 ppb	PCA FDA	12 PCs	>96%
Chu et al. [9]	1000–2500 nm	<20 ppb, 20–100 ppb, and 100 ppb	PCA SVM	5 PCs	82.50%

## Data Availability

The data that support the findings of this study are available upon request from the authors.

## References

[1] Gao J.Y., Ni J.G., Wang D.W., Deng L.M., Li J., Han Z.Z. (2020). Pixel-level aflatoxin detecting in maize based on feature selection and hyperspectral imaging. Spectrochim. Acta Part A Mol. Biomol. Spectrosc..

[2] Teye E., Huang X., Afoakwa N. (2013). Review on the potential use of near infrared spectroscopy (NIRS) for measurement of chemical residues in food. Am. J. Food Sci. Technol..

[3] FDA (2018). Guidance for industry: Action levels for poisonous or deleterious substances in human food and animal feed. Content Current as of September.

[4] Ropodi A.I., Panagou E.Z., Nychas G.J.E. (2016). Data mining derived from food analyses using non-invasive/non-destructive analytical techniques; determination of food authenticity, quality & safety in tandem with computer science disciplines. Trends Food Sci. Technol..

[5] Zhou Q., Huang W.Q., Fan S.X., Zhao F., Liang D., Tian X. (2020). Non-destructive discrimination of the variety of sweet maize seeds based on hyperspectral image coupled with wavelength selection algorithm. Infrared Phys. Technol..

[6] Han Z., Gao J. (2019). Pixel-level aflatoxin detecting based on deep learning and hyperspectral imaging. Comput. Electron. Agric..

[7] Jiang J., Qiao X., He R. (2016). Use of Near-Infrared hyperspectral images to identify moldy peanuts. J. Food Eng..

[8] Bertani F.R., Businaro L., Gambacorta L., Mencattini A., Brenda D., Di Giuseppe D., De Ninno A., Solfrizzo M., Martinelli E., Gerardino A. (2020). Optical detection of aflatoxins B in grained almonds using fluorescence spectroscopy and machine learning algorithms. Food Control.

[9] Chu X., Wang W., Yoon S.C., Ni X., Heitschmidt G.W. (2017). Detection of aflatoxin B-1 (AFB(1)) in individual maize kernels using short wave infrared (SWIR) hyperspectral imaging. Biosyst. Eng..

[10] Zhu F., Yao H., Hruska Z., Kincaid R., Brown R.L., Bhatnagar D., Cleveland T.E. (2016). Integration of fluorescence and reflectance visible near-infrared (VNIR) hyperspectral images for detection of aflatoxins in corn kernels. Trans. Asabe.

[11] Tao F., Yao H., Hruska Z., Liu Y., Rajasekaran K., Bhatnagar D. (2019). Detection of aflatoxin B1 on corn kernel surfaces using visible-near infrared spectra. J. Near Infrared Spectrosc..

[12] Chakraborty S.K., Mahanti N.K., Mansuri S.M., Tripathi M.K., Kotwaliwale N., Jayas D.S. (2021). Non-destructive classification and prediction of aflatoxin-B1 concentration in maize kernels using Vis–NIR (400–1000 nm) hyperspectral imaging. J. Food Sci. Technol..

[13] Kimuli D., Wang W., Lawrence K.C., Yoon S.C., Ni X., Heitschmidt G.W. (2018). Utilisation of visible/near-infrared hyperspectral images to classify aflatoxin B1 contaminated maize kernels. Biosyst. Eng..

[14] Li B., Beveridge P., O’Hare W.T., Islam M. (2013). The age estimation of blood stains up to 30 days old using visible wavelength hyperspectral image analysis and linear discriminant analysis. Sci. Justice.

[15] Garcia-Allende P.B., Conde O.M., Mirapeix J., Cobo A., Lopez-Higuera J.M. (2008). Quality control of industrial processes by combining a hyperspectral sensor and Fisher’s linear discriminant analysis. Sens. Actuators B Chem..

[16] Xia C., Yang S., Huang M., Zhu Q., Guo Y., Qin J. (2019). Maize seed classification using hyperspectral image coupled with multi-linear discriminant analysis. Infrared Phys. Technol..

[17] Han Z., Deng L. (2018). Application driven key wavelengths mining method for aflatoxin detection using hyperspectral data. Comput. Electron. Agric..

[18] Wang W., Heitschmidt G.W., Windham W.R., Feldner P., Ni X., Chu X. (2015). Feasibility of Detecting Aflatoxin B-1 on Inoculated Maize Kernels Surface using Vis/NIR Hyperspectral Imaging. J. Food Sci..

[19] Hove M., Van Poucke C., Njumbe-Ediage E., Nyanga L.K., De Saeger S. (2016). Review on the natural co-occurrence of AFB1 and FB1 in maize and the combined toxicity of AFB1 and FB1. Food Control.

[20] Li J., Chen L., Huang W. (2018). Detection of early bruises on peaches (*Amygdalus persica* L.) using hyperspectral imaging coupled with improved watershed segmentation algorithm. Postharvest Biol. Technol..

[21] Li J., Luo W., Wang Z., Fan S. (2019). Early detection of decay on apples using hyperspectral reflectance imaging combining both principal component analysis and improved watershed segmentation method. Postharvest Biol. Technol..

[22] Kowkabi F., Keshavarz A. (2019). Using spectral Geodesic and spatial Euclidean weights of neighbourhood pixels for hyperspectral Endmember Extraction preprocessing. ISPRS J. Photogramm. Remote Sens..

[23] Savitzky A., Golay M.J. (1964). Smoothing and differentiation of data by simplified least squares procedures. Anal. Chem..

[24] Wang H., Peng J., Xie C., Bao Y., He Y. (2015). Fruit quality evaluation using spectroscopy technology: A review. Sensors.

[25] Kemsley E.K. (1996). Discriminant analysis of high-dimensional data:a comparison of principal components analysis and partial least squares data reduction methods. Chemom. Intell. Lab. Syst..

[26] Wang W., Heitschmidt G.W., Ni X., Windham W.R., Hawkins S., Chu X. (2014). Identification of aflatoxin B-1 on maize kernel surfaces using hyperspectral imaging. Food Control.

[27] Romero I. (2010). PCA-based noise reduction in ambulatory ECGs. IEEE Comput. Cardiol..

[28] Sun P., Bao K., Li H., Li F., Wang X., Cao L., Li G., Zhou Q., Tang H., Bao M. (2018). An efficient classification method for fuel and crude oil types based on m/z 256 mass chromatography by COW-PCA-LDA. Fuel.

[29] Zhou C., Wang L., Zhang Q., Wei X. (2013). Face recognition based on PCA image reconstruction and LDA. Optik.

[30] Zuo W., Zhang H., Zhang D., Wang K. (2010). Post-processed LDA for face and palmprint recognition: What is the rationale. Signal Process..

[31] Jiao L., Wu H., Bie R., Umek A., Kos A. (2018). Towards Real-Time Multi-Sensor Golf Swing Classification Using Deep CNNs. J. Database Manag..

[32] Ben-David A. (2008). About the relationship between ROC curves and Cohen’s kappa. Eng. Appl. Artif. Intell..

[33] Chen H., Pan T., Chen J., Lu Q. (2011). Waveband selection for NIR spectroscopy analysis of soil organic matter based on SG smoothing and MWPLS methods. Chemom. Intell. Lab. Syst..

[34] Berardo N., Pisacane V., Battilani P., Scandolara A., Pietri A., Marocco A. (2005). Rapid detection of kernel rots and mycotoxins in maize by near-infrared reflectance spectroscopy. J. Agric. Food Chem..

[35] Fernandez-Ibanez V., Soldado A., Martinez-Fernandez A., de la Roza-Delgado B. (2009). Application of near infrared spectroscopy for rapid detection of aflatoxin B1 in maize and barley as analytical quality assessment. Food Chem..

[36] Kandpal L.M., Lee S., Kim M.S., Bae H., Cho B.K. (2015). Short wave infrared (SWIR) hyperspectral imaging technique for examination of aflatoxin B-1 (AFB(1)) on corn kernels. Food Control.

[37] Praveen Kumar D., Amgoth T., Annavarapu C.S.R. (2019). Machine learning algorithms for wireless sensor networks: A survey. Inf. Fusion.

[38] Han Z., Deng L. (2020). Aflatoxin contaminated degree detection by hyperspectral data using band index. Food Chem. Toxicol..

